# Risk factors for mortality among multi-drug resistant tuberculosis patients in treatment follow-up centers, eastern Ethiopia: a retrospective follow-up study

**DOI:** 10.11604/pamj.2022.43.78.31929

**Published:** 2022-10-13

**Authors:** Kedir Urgesa Bofe

**Affiliations:** 1 Haramaya University, College of Health and Medical Sciences, School of Medical Laboratory Sciences, Harar, Ethiopia

**Keywords:** Multidrug resistance tuberculosis, mortality, eastern Ethiopia

## Abstract

**Introduction:**

multi-drug resistance tuberculosis (MDR-TB) is associated with an increased risk of mortality among patients on treatment. Ethiopia is the countries with the high MDR-TB burden. This study aimed to determine the extent of mortality and associated factors among MDR-TB patients on treatment in eastern Ethiopia.

**Methods:**

all completely documented data from June 2014 to January 2017 on MDR-TB patients were extracted from patients´ records, between December 2016 and January 2017, in Dader and Dire Dawa MDR treatment follow-up centers. Sociodemographic characteristics, clinical characteristics of MDR-TB patients, and treatment outcomes were extracted from the patients' records. Descriptive statistical methods were used to characterize the sociodemographic variables and the extent of mortality. Binary and multivariable logistic regression analyses were performed to assess factors associated with mortality using an adjusted odds ratio (AOR) at 95% confidence interval (CI). Statistical significance was considered at a P-value of less than 0.05.

**Results:**

among 150 MDR-TB patients, 60.7% of them were males and their mean age was 30.34 + 1.06 years. In this study, the overall mortality rate was 11.3% (95% CI= 6.74-17.52). Previous history of ant-TB treatment (AOR=6.7, 95% CI: 1.59 - 17.15, P= 0.019), hospitalization (AOR=19.55, 95% CI: 6.23-43.37, P=0.001), and human immunodeficiency virus (HIV) coinfection (AOR=6.3, 95% CI: 2.98- 14.0, P= 0.008) were significantly associated with this mortality.

**Conclusion:**

considerably high rate of mortality among MDR-TB patients on treatment highlights the need for more efforts in TB treatment and monitoring the program to limit mortality among MDR-TB patients in the study settings.

## Introduction

Multidrug-resistant tuberculosis (MDR-TB) is a TB that is resistant to at least the two most potent anti-TB drugs (isoniazid and rifampicin) [1,2]. It is a man-made problem; primarily because of human error due to inadequate supply management and anti-TB drugs, and improper treatment [3]. There are signaling reports of an increasing trend of MDR-TB in different parts of the world [4]. Globally, the number of MDR-TB patients detected and notified in 2019, showed a 10% increment from the 2018 report [5]. Particularly, the occurrence of MDR-TB is a major health threat to communities in developing countries [6]. Among TB patients on treatment, MDR-TB is associated with an increased risk of mortality [7,8]. Besides, treating MDR-TB is very complex, takes a longer time, and is associated with poor treatment outcomes [8,9]. Globally, approximately about 20% of TB patients on multi-drug treatment die during treatment every year [10] and the burden of mortality among MDR-TB in resource-limited countries is higher [10,11]. Drug resistance TB patients´ mortality from treatment and care is also a growing public health concern in Ethiopia and the country is among the high MDR-TB burden countries [12-14]. In the country, the prevalence of MDR-TB among re-treated patients and newly diagnosed patients was 15% and 2%, respectively [15] showing MDR-TB is a serious public health problem. Different factors have been shown associated with mortality among MDR-TB patients [16]. Of these, HIV coinfection with TB was the major risk factor for mortality among patients under multi-drug treatment [11,13,16]. Although studies have been shown increased trends of MDR-TB in Ethiopia, little is known regarding the extent of mortality among MDR-TB patients and associated factors in the eastern part of the country. Thus, this study aimed to determine the extent of mortality among MDR-TB and assess factors associated with this mortality in this study setting. Subsequently, health policymakers and other stalk holders could have evidence to control the incidence of MDR and its poor treatment outcomes. Overall, the finding of this study would be an important input to the national/regional TB control program to take evidence-based measures.

## Methods

**Study design and area:** a retrospective cross-sectional study was conducted in Dader and Dire Dawa MDR-TB treatment follow-up centers (TFCs) between December 1^st^, 2016, and January 10^th^, 2017. Dader MDR-TB TFC is one of the 17 MDR TFCs in the Oromia regional state and is located in the East Hararghe Zone of the Oromia regional state, eastern Ethiopia. It is located about 430 Km east of Addis Ababa the capital city of the country. Dire Dawa MDR-TB TFC is the only MDR-TB treatment follow-up center in Dire Dawa City Administration, eastern Ethiopia. It is located about 500 km east of Addis Ababa.

**Study population:** a document review was conducted among all cases of MDR-TB patients who were registered and started treatment during the treatment period from June 1^st^, 2014, to January 10^th^, 2017. All patients who had been on the directly observed therapy (DOTS) regimen of the MDR-TB drug at TFCs and completed treatment charts in the study area were considered. This study included TB patients resistant to Rifampicin and isoniazid and having a complete record of treatment outcomes. The MDR TB patients with an incomplete record on the TB registration book and on treatment at the end of the study were excluded.

**Sample size and sampling:** Epi info version 7.2.2.6 was used to calculate the sample size using a single population formula with a 95% confidence interval (CI), at 5% margin of error, and taking the proportion of mortality among the MDR-TB patients from previous studies with 10.8% [17] and the minimum sample size was 148. Accordingly, during the study period, there are 150 with a complete record. Hence, all MDR-TB patients' registration charts with complete information were included consecutively.

**Data collection:** a structured checklist adopted from national guidelines was used to extract data from patients´ cards and the MDR-TB registration book. In this regard data on sociodemographic characteristics of MDR-TB patients (age, sex, educational status, and address), treatment outcome (mortality), treatment period (intensive and continuous phase), types of resistance, and co-morbid illness were included. The patients´ identification numbers were used to generate the necessary sample from the records of the hospitals for extracting data. Three trained data collectors (nurses from the TB clinic) and supervisors (TB focal persons) were involved in the data collection.

### Study variables

**Dependent variables:** mortality (death) was considered as the outcome variable. Patients who were cured, treatment completed, treatment failed, lost to follow-up, and transferred out were considered as censored.

**Independent variables:** the independent variables were sociodemographic characteristics (sex, age, residence, and educational status), smoking and alcohol drinking behaviors, clinical characteristics including HIV coinfection, diabetes mellitus (DM) co-morbidity, MDR-TB category, previous TB treatments, hospitalization, and TB type.

**Data analysis:** data were entered in EpiData 3.1 (Odense, Denmark) and analyzed using STATA 14 (StatCorp LP., College Station, Texas, US). After the data entry, the collected information was cross-checked with the data collection forms. Descriptive statistical methods were used to summarize the sociodemographic characteristics of the study participants. The Chi-square test was performed to compare categorical variables. Bivariate and multivariable logistic regression analyses were performed to assess factors associated with mortality. A p-value of <0.25 in the univariate analysis, or clinically or epidemiologically relevance of the variables were the criteria for including variables in the multivariable analysis. In multivariable analysis, a P < 0.05 was used for statistical significance at 95% confidence level.

**Ethical consideration:** the study protocol was reviewed and approved by Haramaya University, College of Health and Medical Sciences Institutional Health Research Ethics Review Committee (IHRERC) (reference No.02/2015). Permission was obtained from the respective Regional Health Bureau, district health offices, and the head of the Hospital and MDR-TB treatment center. As this was conducted on anonymized secondary data, patient informed consent was waived. To ensure their confidentiality, study participants were represented by codes.

### Definitions

**MDR-TB:** it is a TB that does not respond to at least isoniazid and rifampicin, the two most powerful anti-TB drugs [1,2].

**Dots:** is an acronym for directly observed therapy short course and a method of drug administration in which a healthcare professional watches as a person takes each dose of medication [18].

**Co-infection:** the presence of HIV among MDR-TB infected individual.

**TB treatment outcome:** the final known status of a TB patient who was started on anti-TB treatment [19].

**Mortality (death):** a patient who died for any reason during the course of treatment.

**Treatment success:** the sum of patients who were declared 'cured´ and those who had 'completed´ treatment [19].

## Results

**Sociodemographic characteristics of study participants:** data from a total of 150 MDR-TB patients (120 from Dire Dawa MDR-TB treatment center and 30 from Dader Hospital MDR-TB TFCs) were reviewed. The mean (+SD) age of the participants was 30.34 (±1.06) years. The majority of participants were male 91 (60.7%) and urban residents 114 (76.0 %), respectively. Regarding participants' educational status, 30 (20 %) of them had not attended formal education (Table 1).

**Table 1:** demographic and clinical characteristics of MDR- TB patients, TB patients, in eastern parts of Ethiopia, 2017

Variable	Variable category	Number	Percent
Name of health facility	Dire Dawa	120	80.0
	Dader	30	20.0
Gender	Male	91	60.7
	Female	59	39.3
Age category	< 15 years	12	8.0
	15 -44years	106	70.67
	45-64 years	22	14.67
	>65	1	6.67
Residence	Urban	114	76.0
	Rural	36	24.0
Level of education	No formal education	30	20.0
	>Read and write	14	9.3
	1-4 grade	13	8.7
	5-8 grade	49	32.7
	9-12 grade	36	24.0
	Higher Education	8	5.3
Patient category	New	46	30.67
	Retreated	102	68.00
	Return after default	2	1.33>
Types of TB	Smear positive pulmonary	148	98.67
	Smear negative, pulmonary	2	1.33
HIV Co-infection	Yes	26	17.33
	No	124<	82.67
DM status	Yes	4	2.67
	No	146	97.33
Housing condition	Private	93	62
History of smoking	Rent house	49
	Homeless	6	4.0
	Prisoner	2	1.33
	Yes	7	4.67
History of MDR contact	No	143	96.33
	Yes	6	4.0
History of Alcohol consumption	No	144	96.0
	Yes	29/td>	19.33
History of hospitalization>	No	121	80.67
	Yes	9	5.33

**Clinical characteristics of the study participants:** of the 150 patients, 148 (98.7%) were pulmonary smear-positive patients, 30 (20%) patients had at least one co-morbidity. The most common coinfection was HIV 26/30 (86.7%), followed by DM 4/30 (13.3%). The majority of patients 102 (68.0%) were retreated category, followed by new category 46 (30.67%) and defaulter cases 2 (1.33%) (Table 1).

**Mortality rate among MDR-MTB patients:** the overall mortality rate was 17/150 (11.3%) (95% CI= 6.74-17.52) during the time of the follow-study. The proportion of death reported among urban resident MDR TB patients was significantly higher than among rural MDR TB patients (11.4% vs 11.1%, X^2^=13.89, P=0.001) (Table 2).

**Table 2:** descriptive results of mortality versus demographic factors among MDR-TB patients, in eastern Ethiopia, 2017

Variables	Category	Mortality		X^2^ (p-value)
		Yes, n(%)	No, n(%)	
Health facility	Dire Dawa	15 (12.5)	105 (87.5)	0.813 (0.526)
	Dader	2 (6.7)	28 (93.3)	
Gender	Male	13 (14.3)	78 (85.7)	3.049 (0.081)
	Female	4 (6.8)	55 (93.2)	
Age of the patient	<15 years	0 (0.00)	12 (100.0)	6.0598 (0.109)
	15-44 years	13 (12.5)	93 (87.5)	
	45-64years	4 (18.2)	18 (81.8)	
	65	0 (0.00)	10 (100)	
Residence	Urban	13 (11.4)	101 (88.6)	13.89 (0.000)*
	Rural	4 (11.1)	32 (88.9)	
Level of education	No formal education	3 (10.0 )	27 (90.0)	8.180 (0.085)
	Read and write	2 (14.3)	12 (85.7)	
	1-4 grade	0(0.0)	13 (100.0)	
	5-8 grade	5(10.2)	44 (89.8)	
	9-12 grade	7 (19.4 )	29 (80.6)	
	Higher Education	0(0.0)	8 (100.0)	

**Factors associated with mortality:** the bi-variable logistic regression showed that being rural residence (crude odds' ratio (COR)= 0.084.95% CI: 0,02-0.34, P= 0.001), HIV coinfection (COR=6.1,95%CI: 5.06-11.8, P=0.001), history of hospitalization (COR=6.25.95% CI: 1.03-23.12, P=0.001), previous TB treatment (COR = 3.2.95% CI: 5.27-18.42,P =0.000), and alcohol consumption behaviors (COR= 0.14,95%CI: 0.02-0.84,P= 0.031) were significantly associated mortality among patients on MDR-TB treatment (Table 3) 3.2(5.27-18.42). However, in the multivariable model, only the patient category with previous anti-TB treatment history (AOR)=6.7, 95% CI: 1.59 - 17.15, P=0.019), history of hospitalization (AOR=19.55, 95% CI: 6.23-43.37, P=0.001), and HIV coinfection (AOR=6.3, 95% CI: 2.98- 13.2, P= 0.008) were identified as independent factors associated with mortality among patients on MDR-TB treatment (Table 3).

**Table 3:** multivariable logistic regression analysis and risk factors of mortality among MDR-TB patients in eastern Ethiopia, 2017

Variable category	Mortality		COR (95%CI)	AOR (95% CI)
**Gender**	Yes	No		
Male	13 (14.3)	78 (85.7)	1	
Female	4 (6.8)	55 (93.2)	0.33 (0.95-1.16)	
**Residence**				
Urban	13 (11.4)	101 (88.6)	1	1
Rural	4 (11.1)	32 (88.9)	0.084 (0.02-0.34)	0.199 (0.165- 2.39)
**Educational level**				
No formal education	3 (10.0 )	27 (90.0)	1	1
Read and write	2 (14.3)	12 (85.7)	5 (0.49-50.83)	1.3(0.035-54.65)
1-4 grade	0(0.0)	13 (100.0)	1.66 (0.12-22.00)	1.53 (0.06-36.05)
5-8 grade	5 (10.2)	44 (89.8)	6 (1.07-33.37)	1.05 (0.07- 13.87 )
9-12 grade	7 (19.4 )	29 (80.6)	8.75 (1.52-50.11)	1.55 (0.100-24.11 )
Higher education	0(0.0)	8 (100%)	1.26 (3.14-12.15)	1.42 (0.08- 6.45)
**Patient category**				
New	3(6.52)	43 (93.48)	1	1
Retreated	13 (12.7)	89 (87.3)	3.2 (5.27-18.42)	6.7 (1.59-17.15)*
Defaulter	1 (50.0)	1 (50.0)	5.2 (0.27-97.61)	2.86 (0.022-373.4)
**History of Mdr contact**				
No	16 (11.2)	127 (88.8)	1	
yes	1 (14.3)	6 (85.7)	0.55 (0.07-4.35)	
**History of smoking**				
yes	16(11.2)	127(88.8)	1	
No	1 (14.3)	6 (85.7)	0.54 (0.13-2.22)	
**Alcohol consumption**				
No	12 (10.0)	109 (90.0)	1)	1
Yes	5 (17.3)	24 (82.7)	0.14 (0.02-0.84)	3.20(0.120-85.35)
**HIV co-infection**				
No	10 (8.6)	109 (91.4)	1	
yes	5 (19.2)	21 (80.8)	6.1 (5.06-11.8)	6.3 (2.98-13.2)*
**Hospitalization**				
No	8 (5.7)	133 (94.3)	1	1
Yes	9 (100.0)	0 (0.0)	6.25 (1.03-23.12)	19.55 (6.23-43.37)*

note: *presence of statistically significant difference (P < 0.05; AOR=adjusted odds ratio, CI= confidence interval, COR= crude odds ratio, **1= reference**

## Discussion

This study abridged the patients´ mortality and its associated factors among MDR-TB patients who were treated at MDR-TB TFCs in eastern Ethiopia. The mortality among MDR-TB patients under treatment in this study setting was 11.3%. This finding concluded that mortality among MDR-TB patients remains significant in these study settings. The mortality rate in this study is comparable with a multicenter observational study conducted in South and Southwest Ethiopia, which reports an 11% death rate among MDR-TB [19]. In addition, a retrospective cohort study from treatment-initiating centers (TICs) in Ethiopia reported a 12.8% death rate among patients with MDR-TB [20]. However, the mortality rate in this study is higher than in studies conducted in different parts of Ethiopia [10,21-23]. The high mortality rate among MDR-TB patients under treatment in this study setting shows a need to strengthen early case detection and proper treatment of drug-susceptible TB based on WHO treatment guidelines to ensure adequate treatment success rates. Hence, early identification of resistance and the use of a properly designed regimen could improve treatment outcomes (cure rate) [18]. On contrary, our5 finding is lower than other studies conducted in different parts of Ethiopia [24-26]. The variation could be recognized as different in the study period and effort made in combating the crisis of MDR-TB in a different region of the country. Studies in Ethiopia identified many factors associated with the risk of mortality among MDR-TB patients [22,24,27]. However, in the present study, only previous history of treatment, hospitalization, and HIV coinfection was found to be independent risk factors of mortality among MDR-TB patients. This variation could be because of the sampling size and age of study participants difference, as most studies exclude TB patients younger than 15 years.

In this study, one in five MDR-TB patients had co-morbidity with chronic illness. Of this, the majority (86.7%) of them were HIV coinfection. Multi-drug resistant tuberculosis patients who were being coinfected with HIV had unsuccessful treatment outcomes (mortality) compared to patients being not coinfected with HIV. This finding is in line with other studies conducted in Ethiopia, which reported HIV coinfection was an important predictor of mortality among MDR-TB patients under treatment [11,22,25,26,28]. This is due to the known fact that HIV coinfection affected the integrity and function of CD4+ cells, which reduced the level of immunity and increased the risk of mortality [21]. This combined effect may be the main reason for the mortality among HIV -coinfected MDR-TB patients. Therefore, persistent integration of TB/HIV services may help in reducing drug-resistant tuberculosis mortality [26]. Hence, screening of MDR-TB for coinfection, in particular for HIV is critical to minimize unsuccessful treatment outcomes and to improve treatment success in coinfected patients. In the present study, a history of hospitalization during follow-up for treatment was found to be an important factor associated with mortality. The finding is in line with the finding that hospital-acquired infection (HAI) TB exposure is associated with one-year TB incidence and TB-related mortality [29]. This explains the fact that, during a prolonged hospital stay, TB patients may develop other HAI that can enhance the complication and result in death. Our study revealed that the mortality was significantly different among MDR-TB patients who were retreated compared to new MDR-TB patients. This is in agreement with the study carried out in different parts of Ethiopian Hospitals that revealed the likelihood of unsuccessful treatment outcomes was more frequent in pretreatment than in newly treated cases [12,15,16,26,28]. This finding indicates that, inadequate regional healthcare strategies in controlling TB and population access to healthcare facilities differences and TB control programs. Hence, proper treatments of drug-susceptible TB patients and improved early case detection of drug-resistant TB are indispensable, to improving TB's successful treatment outcomes in the region.

**Strengths and limitations of the study:** this study provides overall mortality and its associated factors among patients on MDR-TB treatments in TFCs in eastern parts of the country. Nevertheless, the retrospective nature of the data source limited this study from tracking the major causes of death. As the data were obtained from records in public health institutions' medical records and some of the information might not be consistently recorded. Furthermore, Small sample size may affect the finding of this study.

## Conclusion

The mortality rate was considerably higher than the national reports in this study. Re-treatment, hospitalization, and HIV c-infection were independent risk factors of MDR-TB patient mortality. Hence, regional and zonal TB control programs in these study settings should pay due attention to MDR-TB patients with previous anti-TB treatments, a history of hospitalization, and HIV coinfection to limit mortality among these patients. Hence, researchers are recommended to focus on

### What is known about this topic

- The burden of mortality due to MDR-TB in resource-limited countries is higher;
- The prevalence of MDR-TB is increasing in Ethiopia; however, little is known regarding the magnitude of mortality and associated factors among MDR-TB patients in the current study site.

### What this study adds

- This study depicts the extent of mortality among MDR-TB patients on treatment in eastern parts of Ethiopia;
- HIV co-infection, history of hospitalization and previous anti-TB treatments are important factors associated with this mortality in this study setting;
- HIV is the most co-infection occurred in MDR-TB patients.
